# Follow-up of Bernese Mountain dogs and other dogs with serologically diagnosed *Borrelia burgdorferi *infection: What happens to seropositive animals?

**DOI:** 10.1186/1746-6148-5-18

**Published:** 2009-05-08

**Authors:** Bernhard Gerber, Katharina Haug, Simone Eichenberger, Claudia E Reusch, Max M Wittenbrink

**Affiliations:** 1 Clinic for Small Animal Internal Medicine, Vetsuisse Faculty University of Zurich, Winterthurstrasse 260, 8057 Zurich, Switzerland; 2 Institute of Veterinary Bacteriology, Vetsuisse Faculty University of Zurich, Winterthurstrasse 270, 8057 Zurich, Switzerland

## Abstract

**Background:**

Data on the long-term outcome of *B. burgdorferi *infections in adult dogs are sparse. The aim of the present study was to investigate whether Bernese Mountain dogs with serological evidence of natural *B. burgdorferi *infection more often develop signs such as lameness, azotemia or proteinuria during a follow-up period of 2.5 to 3.0 years. Seropositive Bernese Mountain dogs were compared to seronegative Bernese Mountain dogs and to seropositive and seronegative control dogs of other breeds.

Dogs included in a previous study on the prevalence of antibodies against *B. burgdorferi *in Bernese Mountain dogs were re-evaluated. Antibodies against *B. burgdorferi *were determined using an ELISA with a whole-cell sonicate as antigen and results were confirmed using a Western blot assay.

**Results:**

Fifty-three Bernese Mountain dogs and 30 control dogs were re-evaluated. Re-evaluation was performed between 2.5 and 3.0 years (median 2.7 years) after the first assessment.

The age of the dogs at the second evaluation ranged from 3 to 11 years (median 6 years). There were no significant differences with regard to poor general condition or lameness between the first and the second evaluation.

At the first evaluation 22 (42%) of the Bernese Mountain dogs and 11 (37%) of the control dogs were considered positive for antibodies against *B. burgdorferi*. At the second evaluation 25 (47%) of the Bernese Mountain dogs and 12 (40%) of the control dogs were considered positive; 69% of the dogs showed the same serological result at both examinations and 31% were seroconverted or seroreverted. During the first examination, azotemia was diagnosed in 6 Bernese Mountain dogs and during the second examination in 11 Bernese Mountain dogs. No control dogs had azotemia in this study. In seropositive dogs there was no increase in lameness or signs of renal disease over time.

**Conclusion:**

It may be concluded that antibodies against *B. burgdorferi *determined by whole cell ELISA and confirmed by Western blot were neither associated with the development of lameness nor with signs of renal disease like azotemia or proteinuria in dogs observed over a period of 2.5 to 3.0 years.

## Background

Prior serologic surveys have shown that long-term persistence of *B. burgdorferi*-specific antibodies in dogs may occur. However, data on the long-term outcome of said infections in adult dogs are sparse [1,2]. In experimentally infected young beagles recurrent lameness was observed up to 280 days post infection [3]. In a post mortem study lymphadenopathy was found in the area of tick attachment and there were microscopical signs of inflammation in synovial membranes, joint capsules and tendon sheets after exposure to infected ticks [4]. Further observation lent some support to the assumption that canine *B. burgdorferi *infection is also related to renal disease [5,6]. The term "Lyme nephritis" was introduced to describe a renal disease with specific renal histopathologic lesions including immune-mediated glomerulonephritis, tubular necrosis and interstitial nephritis in dogs in which antibodies against *B. burgdorferi *were detected [6]. Considering the proposed pathophysiology of Lyme nephritis to be an immune-mediated glomerular disease [6,7] one would expect a progressive course with dogs developing disease after chronic infection. However, experimental infection of young Beagle dogs with *B. burgdorferi *sensu stricto gave no pathologic indications of glomerular disease for up to 581 days after experimental exposure to infected ticks [4]. It was speculated that under natural conditions age, breed or the pathological features of the borrelial agent might influence the development of Lyme nephritis. However, there are as yet no data on the long-term clinical outcome of natural *B. burgdorferi *infections in dogs. Recent studies partly support an interrelation between seroprevalence of *B. burgdorferi *and genetic predisposition for increased susceptibility to borrelial infections in Bernese Mountain dogs [8].

The aim of the present study was to investigate whether Bernese Mountain dogs with serological evidence of natural *B. burgdorferi *infection more often develop signs such as lameness, proteinuria or azotemia during a follow-up period of 2.5 to 3.0 years. Seropositive Bernese Mountain dogs were compared to seronegative Bernese Mountain dogs, and to seropositive and seronegative control dogs of other breeds.

## Methods

### Samples and dogs

Dogs included in a previous study on the prevalence of *B. burgdorferi *in Bernese Mountain dogs were re-evaluated after 2.5 years (899 days), and after 3.0 years (1113 days) (median 2.7 years (992 days)) [8]. This study focussed on including similar numbers of dogs of the following groups: Bernese Mountain dogs in which antibodies against *B. burgdorferi *were diagnosed, Bernese Mountain dogs in which no antibodies against *B. burgdorferi *were detected, control dogs (long haired large breed dogs but not Bernese Mountain dogs) in which antibodies against *B. burgdorferi *were diagnosed, and control dogs in which no antibodies against *B. burgdorferi were *detected. For the first study dogs were sampled between July 2002 and April 2003. There were 160 Bernese Mountain dogs and 62 control dogs. Dogs were re-evaluated between June and October 2005. The owners of 82 Bernese Mountain dogs and 62 control dogs evaluated in the previous study were contacted a second time in order to evaluate the response over time of clinical and laboratory parameters. For 29 Bernese Mountain dogs (35%) and 32 control dogs (52%) no second examination was possible because the dogs were either dead (25 Bernese Mountain dogs and 14 control dogs) or the owners could not be reached a second time, or were unwilling to participate again (4 Bernese Mountain dogs and 18 control dogs). The health status of the dogs was assessed using a questionnaire filled in by the owners. Answers relating to general health and lameness were compared between the first and the second examination to assess possible consequences of a *B. burgdorferi *infection. Owners were asked to judge if the general health of their dog was normal or abnormal, and if their dogs were lame or not at the time of the second evaluation. At both time-points routine laboratory tests of blood and urine samples were performed. For serologic testing samples were frozen at minus 80°Celsius.

### Haematology and serum biochemistry

Laboratory tests included a complete blood count (CBC) and a serum biochemical analysis containing determination of bilirubin, glucose, urea, creatinine, total protein, albumin, cholesterol, sodium, potassium, chloride, calcium and phosphorus concentrations; as well as the activity of alkaline phosphatase, alanine transferase, aspartate transferase and amylase. Hematocrit, urea, creatinine, total protein and albumin values during the first and the second examination were compared to assess renal function and function of the filtration barrier. Dogs were deemed azotemic if creatinine was above 125 μmol/L and/or if urea was above 9.4 mmol/L.

### Urinalysis

Urinalysis consisted of a urine dip stick (Combur-Test, Roche Diagnostics GmbH, Mannheim Germany), microscopic examination of urine sediment, and determination of urine specific gravity. Results of urine protein measurements were considered only if fewer than 5 leukocytes per 400× field were counted in the urine sediment. The results of the protein measurement with the dip stick were recorded as negative or 1+ to 3+ positive. The urine protein-to-creatinine ratio (UPC) was measured on a Cobas-Integra analyzer (Roche, Rotkreuz, Switzerland). A difference in UPC between the first and the second examination was considered significant if it was 80% or more of the first value [9]. Microalbuminuria was measured by a commercial rapid immunoassay for canine microalbuminuria (E.R.D.-Screen™-Test, HESKA^®^, Fribourg, Switzerland). Results were interpreted as negative, low-positive, medium-positive or high-positive according to the manufacturer's instructions. Urine specific gravity and results of the measurements of protein in urine were compared between the first and the second examination. If the dogs were azotemic, the azotemia was considered renal if the urine specific gravity was below 1.030.

### Serologic testing

Serologic testing of serum stored at minus 80°C was performed. Serologic tests of the samples of the first and the second evaluation were performed together. An enzyme-linked immunosorbent assay (ELISA) for the detection of antibodies against *B. burgdorferi *sensu lato was performed for all dogs according to established protocols as described previously [8,10].

Briefly, a whole-cell sonicate of *B. burgdorferi *sensu stricto reference strain B31 (ATCC 35210), *B. garinii *N34, *B. afzelii *VS 461 and *B. valaisiana *VS 116 was used as antigen. Prior to serological testing serum samples were absorbed with a heterologous sorbant of washed formalin inactivated whole cells of *Escherichia coli*, *Salmonella typhimurium*, *Brachispira hyodysenteriae*, *Bacillus subtilis *and a total of ten serovars of *Leptospira *(*L.*) *interrogans *and *L. borgepetersenii*, respectively.

For each plate positive and negative control samples were applied. The cut-off was calculated from the serial measurement of the negative controls.

Western blot examinations to detect antibodies against *B. burgdorferi *were performed using a commercial test kit modified for dogs (Virion Ltd., Rüschlikon, Switzerland). Tests were performed according to the manufacturer's instructions and consisted of Western blot strips with defined partial antigens of *B. burgdorferi *sensu stricto and *B. afzelii*. Western blot results were interpreted according to criteria recommended for European species of *B. burgdorferi *sensu lato [11]. Samples were called positive if bands at the level of the partial antigens p100, p58, OspC, p21 or wb18 were identified or if at least two bands at the level of the partial antigens p45, bmpa und wb30 were present. Bands at the level of the partial antigens OspB, OspA, OspD, wb22 und OspE were considered unspecific.

Dogs were considered to have antibodies against *B. burgdorferi *if both the ELISA and the Western blot were positive.

### Statistical analysis

Data was recorded and analyzed using a commercial computer program (Statistical Package for the Social Sciences for Windows version 11, SPSS Inc, Chicago Illinois, USA). Data of the first and the second examination was compared using the Wilcoxon signed rank test for ordinal data and McNemar's change test for nominal data. Additionally, to compare age between dogs with and without poor general condition and between dogs with and without lameness the Mann-Whitney U test was used. For dogs re-evaluated after the first examination and those not re-evaluated the Mann-Whitney U test was also used for the comparison of hematocrit, urea, creatinine, protein, albumin, urine specific gravity and urine protein-to creatinine ratio. The Fisher's exact test was used to compare the occurrence of lameness between dogs re-evaluated a second time and those not re-evaluated. Differences were considered significant at P < 0.05. Bonferroni correction was applied in the comparison of haematology, serum chemistry and urine parameters.

## Results

Fifty-three Bernese Mountain dogs and 30 control dogs were re-evaluated. The age of the dogs at the time of the second evaluation ranged from 3 to 11 years (median 6 years). There were 23 male and 60 female dogs.

### Antibodies against *B. burgdorferi*

At the first evaluation, 22 (42%) of the Bernese Mountain dogs and 11 (37%) of the control dogs were considered positive for antibodies against *B. burgdorferi *(Table 1). At the second evaluation, 25 (47%) of the Bernese Mountain dogs and 12 (40%) of the control dogs were considered positive.

**Table 1:** Antibodies against *B. burgdorferi *in Bernese Mountain dogs and control dogs at the first evaluation and after a median of 2.7 years.

	First evaluation			
	
	Bernese Mountain dogs (N = 53)		Control dogs (N = 30)	
	
	*B. burgdorferi *serology			
	
Serology result at the second evaluation	positive (N = 22)	negative (N = 31)	positive (N = 11)	negative (N = 19)
remained negative		21 (68%)		14 (74%)

remained positive	15 (68%)		7 (64%)	

seroconverted*		7 (32%)		4 (36%)

seroreverted**	10 (32%)		5 (26%)	

*seroconversion: Serologic test for antibodies against *B. burgdorferi *was negative in the first evaluation and changed to positive in the second.

**seroreversion serologic test for antibodies against *B. burgdorferi *was positive in the first evaluation and changed to negative in the second.

Of the Bernese Mountain dogs, 36 (68%) had the same serological results at both examinations and 17 (32%) either seroconverted or seroreverted. Of the control dogs, 21 (70%) had the same serological result at both examinations and 9 (30%) either seroconverted or seroreverted. There was no significant change in serological results between the first and the second evaluation in either group.

### Answers to questionnaire

All dogs were considered healthy by their owners at the time of the first evaluation. At the second evaluation, 8 owners stated that the general condition of their dogs had detoriated (Table 2). Of these dogs 1 Bernese Mountain dog and 2 control dogs were seropositive for *B. burgdorferi *in both examinations, 2 control dogs were negative in both examinations, 1 Bernese Mountain dog seroconverted and 1 Bernese Mountain dog and 1 control dog seroreverted. Three of the eight dogs also showed lameness at the time of the second evaluation. (2 seroreverted and 1 remained seropositive). There were no significant differences in the occurrence of a poor general condition between the first and the second evaluation. However, dogs with a poor general condition were significantly older than those with a good general condition at the time of the second evaluation (age 8 to 11 years, median 10 years versus 3 to 10 years, median 6 years; P < 0.001).

**Table 2:** General condition and lameness in Bernese Mountain dogs and control dogs at the first examination and after a median of 2.7 years.

			**Bernese Mountain dogs (N = 53)**		**Control dogs (N = 30)**	
			
			Result of *B. burgdorferi *serology at the first examination			
			
			positive (N = 22)	negative (N = 31)	positive (N = 11)	negative (N = 19)
**General condition**	1^st ^ex.	normal	21	31	11	18
		poor				
	
	2^nd ^ex.	normal	19	30	8	16
		poor	2	1	3	2
			[P = 0.5]*	[P = 1.0]	[P = 0.25]	[P = 0.5]

**Lameness**	1^st ^ex.	no	21	30	10	15
		yes		1	1	3
	
	2^nd ^ex.	no	17	30	9	15
		yes	4	1	2	3
			[P = 0.125]	[P = 1.0]	[P = 1.0]	[P = 1.0]

ex. = examination

*P resulting from comparison between first and second evaluation

Five owners reported lameness in their dogs at the first examination and 10 owners reported lameness at the second evaluation (Table 1). Of the 10 dogs lame at the second evaluation, poor general condition was reported for 3 (2 seroreverted and 1 remained seropositive). Four of the dogs that were lame at the first evaluation were still lame at the second evaluation, whilst 1 seronegative Bernese Mountain dog was no longer lame. Six dogs were newly lame at the second evaluation (5 Bernese Mountain dogs and 1 control dog). Five of these dogs showed antibodies against *B. burgdorferi *at the first evaluation whilst 1 Bernese Mountain dog proved negative. However, 2 Bernese Mountain dogs seroreverted and were negative at the second evaluation, whilst the other dogs kept their serological status. There was no significant difference in the occurrence of lameness between the first and the second evaluation in all groups. Dogs with lameness at the second evaluation were significantly older than the dogs that showed no lameness (6 to 11 years, median 8 years versus 3 to 10 years, median 6 years; P < 0.005).

### Results of laboratory analysis

Results of the laboratory tests and urinalysis are shown in Table 3. At the first examination, 6 Bernese Mountain dogs were azotemic (creatinine 95–155 μmol/L, median 109 μmol/L; urea 7.1–13.1 mmol/L, median 10.1 mmol/L). Renal azotemia was considered a possibility in 5 of these dogs. At the second examination 11 Bernese Mountain dogs were azotemic (creatinine 82–260 μmol/L, median 130 μmol/L; urea 6.5–15.1 mmol/L, median 10.0 mmol/L). In 5 of these dogs renal azotemia was considered possible. No control dogs were azotemic at either examination. Of the 6 Bernese Mountain dogs which were azotemic at the first examination 2 were non azotemic at the second evaluation whilst 4 remained azotemic. At the first examination all azotemic dogs were negative for antibodies against *B. burgdorferi*. At the second examination, 6 of the 11 dogs with azotemia were positive for antibodies against *B. burgdorferi*.

**Table 3:** Range and median of hematology, serum chemistry and urine parameters in Bernese Mountain dogs and control dogs at the first examination and after a median of 2.7 years.

			**Bernese Mountain dogs (N = 53)**		**Control dogs (N = 30)**	
			
			Result of *B. burgdorferi *serology at the first examination			
			
Parameters	Reference range		positive (N = 22)	negative (N = 31)	positive (N = 11)	negative (N = 19)
**Hematocrit **[%]	42–55	1^st ^ex.	41–63 (51)	41–60 (50)	40–57 (51)	43–54 (48)
		2^nd ^ex.	36–54 (46)	36–54 (46)	34–49 (44)	41–55 (47)
			[P < 0.001]*	[P < 0.001]*	[P = 0.004]*	[P = 0.06]

**Urea **[mmol/L]	3.8–9.4	1^st ^ex.	3.1–8.9 (6.7)	4.0–13.1 (6.5)	3.8–7.7 (5.6)	3.4–8.6 (5.6)
		2^nd ^ex.	3.9–13.7 (6.5)	3.6–15.1 (6.9)	2.8–6.4 (4.8)	1.7–6.7 (4.2)
			[P = 0.73]	[P = 0.34]	[P = 0.21]	[P = 0.001]*

**Creatinine **[μmol/L]	64–125	1^st ^ex.	80–120 (102)	74–155 (100)	72–119 (87)	66–114 (82)
		2^nd ^ex.	64–138 (108)	65–260 (99)	59–110 (78)	52–107 (72)
			[P = 0.85]	[P = 0.96]	[P = 0.003]*	[P < 0.001]*

**Protein **[g/L]	56–71	1^st ^ex.	58–74 (64)	43–74 (63)	53–65 (61)	50–67 (61)
		2^nd ^ex.	53–70 (64)	55–73 (64)	59–69 (61)	58–74 (63)
			[P = 0.77]	[P = 0.002]*	[P = 0.28]	[P = 0.02]

**Albumin **[g/L]	29–37	1^st ^ex.	25–33 (30)	21–34 (30)	28–35 (32)	28–35 (31)
		2^nd ^ex.	22–36 (33)	28–38 (35)	30–40 (33)	30–40 (35)
			[P = 0.001]*	[P < 0.001]*	[P = 0.03]	[P < 0.001]*

**Urine specific gravity**		1^st ^ex.	1.010–1.049 (1.030)	1.006–1.050 (1.028)	1.011–1.048 (1.021)	1.007–1.048 (1.027)
		2^nd ^ex.	1.006–1.050 (1.030)	1.007–1.050 (1.034)	1.012–1.046 (1.023)	1.006–1.042 (1.026)
			[P = 0.21]	[P = 0.27]	[P = 0.81]	[P = 0.41]

**Urine protein-to-creatinine ratio **(UPC)		1^st ^ex.	0.06–0.28 (0.11)	0.06–0.42 (0.11)	0.07–0.48 (0.12)	0.08–0.21 (0.11)
		2^nd ^ex.	0.05–0.36 (0.14)	0.06–0.44 (0.10)	0.06–0.92 (0.09)	0.06–0.53 (0.11)
			[P = 0.05]	[P = 0.79]	[P = 0.13]	[P = 0.91]

**Urine protein on dip stick**		1^st ^ex.	15 neg., 3 pos.	21 neg., 5 pos.	9 neg., 2 pos.	11 neg., 6 pos
		2^nd ^ex.	6 neg., 11 pos.	7 neg., 18 pos.	4 neg., 7 pos.	9 neg., 8 pos.
			[P = 0.001]*	[P < 0.001]*	[P = 0.03]	[P = 0.27]

**Micro-albuminuria test**		1^st ^ex.	11 neg., 8 pos.	22 neg., 4 pos.	10 neg.	14 neg., 3 pos.
		2^nd ^ex.	11 neg., 6 pos.	20 neg., 5 pos.	10 neg., 1 pos.	13 neg., 4 pos.
			[P = 0.69]	[P = 0.25]	[P = 1.00]	[P = 1.00]

*Significant difference between first and second examination at P < 0.006 due to Bonferroni correction.

ex. = examination

Six Bernese Mountain dogs and 3 control dogs had a significant increase in UPC between the first and the second examination (80% or more of the first value; 0.06–0.44, median 0.20). Four of the Bernese Mountain dogs and 1 of the control dogs had antibodies against *B. burgdorferi *at the first examination. At the second evaluation one more Bernese Mountain dog was positive. All other dogs kept their serologic status.

Of the 16 dogs with a positive dipstick result in the first examination, 4 were negative in the second examination (1 Bernese Mountain dog and 3 control dogs). A total of 21 Bernese Mountain dogs and 11 control dogs changed from a negative dipstick result in the first examination to a positive result in the second examination (30 dogs 1+ positive and 2+ positive). In 7 of the 32 dogs with a change in the dipstick result positive antibodies against *B. burgdorferi *were detected in the first examination and these dogs remained positive, 13 were negative and remained negative, 6 seroconverted and 6 seroreverted. The difference in occurrence of a positive dipstick result between the first and the second examination was significant in Bernese Mountain dogs but not in control dogs.

Overall, 15 (18%) of the dogs were tested positive against microalbuminuria during the first examination and 16 (19%) during the second test. Three dogs were low-positive in the first examination and tested negative in the second examination. Four dogs were high-positive in both the first and second examination. Seven dogs that were negative during the first examination were positive during the second exmination (5 light positive and 2 medium positive). Of the 7 dogs in which the microalbuminuria test changed from negative to positive, 6 were Bernese Mountain dogs, and 1 was a control dog. In 3 of these 7 dogs antibodies against *B. burgdorferi *were detected during the first examination and remained positive, 3 were negative and remained negative and 1 seroconverted.

### Comparison with dogs not evaluated a second time

Not all dogs evaluated in a previous study [8] were examined a second time. To confirm that a representative number of the dogs evaluated the first time was selected, different parameters of the first examination were compared between these dogs and those that were not re-examined. The only significant difference was found in the hematocrit of seronegative control dogs. The hematocrit was higher in dogs evaluated a second time (P = 0.001), ranging from 43–54% (median 48%) in re-evaluated dogs and from 35–53% (median 44%) in non re-evaluated dogs. Seven of the dogs that were not re-evaluated had a hematocrit below the reference range (42–55%).

## Discussion

The purpose of this study was to re-evaluate similar numbers of Bernese Mountain dogs and control dogs in which antibodies against *B. burgdorferi *had been detected or not detected in a previous study [8]. However, even though all owners of control dogs participating in the first study were re-contacted, we were not able to obtain as high a number of control dogs positive for antibodies against *B. burgdorferi *as Bernese Mountain dogs. The main reason was that owners of control dogs were not willing to participate in the study a second time.

We were not able to obtain sufficient useful information on relevant diseases or treatments of the dogs between the two examinations. Accordingly, the influence of past disease problems on the current status of the dogs could not be evaluated. However, comparing the dogs which were re-evaluated with those that were not re-evaluated indicated that a representative group of dogs from the first study was included.

In dogs that were re-evaluated there were only minor differences between the first and the second examination. For some dogs owners reported a poorer general condition at the second evaluation. The unspecific parameter might indicate non obvious health problems. This was considered important because in human medicine non-specific signs like persistent pain and fatigue are attributed to "chronic Lyme disease", which is unfortunately not a well defined term [12]. However, in most dogs poor general condition could be attributed to age-related changes.

Lameness and fever were the only clinical findings in experimentally infected dogs [13]. For this study it was assumed that lameness would be more common in dogs that were positive for *B. burgdorferi *antibodies than in other dogs. However, this was not the case. There are several explanations for this. Possibly *B. burgdorferi *did not cause disease in these dogs at all, or the disease remained subclinical. In addition, it is known that clinical signs are often intermittent and lameness was assessed only at the time-points of both examinations. The question also remains whether European *B. burgdorferi *strains might cause different or no diseases in dogs. The reports on Lyme disease in dogs originate from the United States, where only *B. burgdorferi *sensu stricto are present whilst in Switzerland *B. garinii *predominates, followed by *B. afzelii *and *B. burgdorferi *sensu stricto [14,15]. Furthermore, even within the species of *B. burgdorferi *sensu stricto different genotypes were shown to cause different disease severities in mice [16]. Still more dogs which were seropositive at the first evaluation were lame at the second evaluation; however, if *B. burgdorferi *was involved in the development of lameness one would expect persistent infection and not a seroreversion. Furthermore, lameness due to other arthropathies is likely in old large breed dogs. Therefore, we come to the same conclusion as Levy and Magnarelli (1992)[17], i.e. that the serologic status of apparently healthy dogs has no value in predicting subsequent lameness.

Seroconversion and seroreversion were similar in both examinations, indicating that the percentage of dogs infected might remain stable over time. This differs from a study in an endemic area, where more dogs seroconverted (24%) than seroreverted (9%) [17]. Different strains or recurrent infection might be the reason for this. Interestingly, in humans exposed to ticks seroreversion seems to be more common than seroconversion [18]. Seropositivity is also influenced by the season in which the samples are collected. OD values were found to be lower during the ticks' quiescent period than during the tick season [19]. For the present study the first sampling was partly conducted during the non-tick season. This might have led to fewer positive samples than during the tick-season.

There was no indication of reduced renal function in the parameters examined. Although serum creatinine was higher in the dogs with antibodies against *B. burgdorferi*, azotemia rarely occurred. Furthermore, there were no differences in serum urea concentrations.

The serum albumin concentration was lower in Bernese Mountain dogs than in control dogs. This could not be attributed to urinary loss since the degree of proteinuria in Bernese Mountain dogs was not different from that in other dogs. This corresponds to earlier studies in which no higher prevalence of proteinuria was found in Bernese Mountain dogs or Labrador Retrievers with antibodies against *B. burgdorferi *[20,21]. The only significant difference with regard to protein excretion in the urine was an increase in positive dipstick results in Bernese Mountain dogs during the second examination. As this was the case in both positive and negative dogs an influence of *B. burgdorferi *infection is unlikely. The slightly higher specific gravity in the group of negative Bernese Mountain dogs might explain some of the changes. Furthermore, most dipstick readings changed from negative to only 1+ positive, a change that is minimal and might even depend on the reader.

The test used to establish microalbuminuria had predicted the development of proteinuria in dogs with X-linked hereditary glomerulopathy [22]. This study did not reveal a difference in microalbuminuria between the first and the second examination. The occurrence of microalbuminuria was similar to another report in which 19% of the healthy dogs showed microalbuminuria [23]. The prevalence of microalbuminuria has been reported to increase with age, but in this study there were minimal increases between the first and the second evaluation even though the dogs were almost three years older [24].

## Conclusion

It may be concluded that antibodies against *B. burgdorferi *determined by whole cell ELISA and confirmed by Western blot were not associated with the development of lameness and signs of renal disease like azotemia or proteinuria in dogs observed over a period of 2.5 to 3.0 years.

## Authors' contributions

BG: Designed the study, analyzed the data and drafted the manuscript

KH: Contributed to the study design, collected the data for the second evaluation and contributed to the manuscript drafting and data interpretation.

SE: Collected and interpreted the data for the first evaluation and contributed to the study design.

CER: Was involved in the study design and coordination and contributed to the critical evaluation and interpretation of the data.

MMW: Performed the serologic tests, was involved in the study design and the drafting of the manuscript.
